# Development, modelling, and pilot testing of a complex intervention to support end-of-life care provided by Danish general practitioners

**DOI:** 10.1186/s12875-018-0774-x

**Published:** 2018-06-20

**Authors:** Anna Kirstine Winthereik, Mette Asbjoern Neergaard, Anders Bonde Jensen, Peter Vedsted

**Affiliations:** 1 Department of Oncology, Aarhus University Hospital, Noerrebrogade 44, 8000 Aarhus C, Denmark; 2 Palliative Care Team, Department of Oncology, Aarhus University Hospital, Noerrebrogade 44, 8000 Aarhus, Denmark; 3 Research Unit for General Practice, Department of Public Health, Aarhus University, Bartholins Allé 2, 8000 Aarhus, Denmark; 4 Department of Clinical Medicine, Aarhus University, Noerrebrogade 44, 8000 Aarhus C, Denmark

**Keywords:** Continuing medical education, Clinical decision support systems, Palliative care, End-of-life care, COPD, Cancer, General practice, Complex intervention, Denmark

## Abstract

**Background:**

Most patients in end-of-life with life-threatening diseases prefer to be cared for and die at home. Nevertheless, the majority die in hospitals. GPs have a pivotal role in providing end-of-life care at patients’ home, and their involvement in the palliative trajectory enhances the patient’s possibility to stay at home. The aim of this study was to develop and pilot-test an intervention consisting of continuing medical education (CME) and electronic decision support (EDS) to support end-of-life care in general practice.

**Methods:**

We developed an intervention in line with the first phases of the guidelines for complex interventions drawn up by the Medical Research Council. Phase 1 involved the development of the intervention including identification of key barriers to provision of end-of-life care for GPs and of facilitators of change. Furthermore the actual modelling of two components: CME meeting and EDS. Phase 2 focused on pilot-testing and intervention assessment by process evaluation.

**Results:**

In phase 1 lack of identification of patients at the end of life and limited palliative knowledge among GPs were identified as barriers. The CME meeting and the EDS were developed. The CME meeting was a four-hour educational meeting performed by GPs and specialists in palliative care. The EDS consisted of two parts: a pop-up window for each patient with palliative needs and a list of all patients with palliative needs in the practice. The pilot testing in phase 2 showed that the CME meeting was performed as intended and 120 (14%) of the GPs in the region attended. The EDS was integrated in existing electronic records but was shut down early for external reasons; 50 (5%) GPs signed up. The pilot-testing demonstrated a need to strengthen the implementation as attending rate was low in the current set-up.

**Conclusion:**

We developed a complex intervention to support GPs in providing end-of-life care. The pilot-test showed general acceptance of the CME meetings. The EDS was shut down early and needs further evaluation before examining the whole intervention in a larger study, where evaluation could be based on patient-related outcomes and impact on end-of-life care.

**Trial registration:**

Clinicaltrials.gov (NCT02050256) January 30, 2014.

**Electronic supplementary material:**

The online version of this article (10.1186/s12875-018-0774-x) contains supplementary material, which is available to authorized users.

## Background

The general practitioner (GP) has a pivotal role in palliative care in most western countries. In particular, when the patient is at home, the GP is the key physician [1] and optimally ensures all aspects of continuity in the illness trajectory [2]. The GP often acts as gatekeeper to specialist treatment [3] and thus has the potential to assume a coordinating role for patients with cancer and other life-threatening disease.

Most patients with terminal illnesses prefer to be cared for and die at home [4, 5]. Nevertheless, the majority of patients in Denmark and many other western countries end up dying in hospitals [4–6]. Involvement of the GP in the palliative trajectory seems to enhance the patient’s possibility to stay and die at home [7–9], but a need for optimising the end-of-life care has been identified among Danish GPs [10]. Furthermore, previous studies have found that cancer patients were more likely to receive palliative care compared to patients suffering from non-malignancies (e.g. COPD and heart failure) [11–13].

Therefore, it is crucial to support and optimise the end-of-life care provided by GPs to both cancer patients and patients suffering from non-malignant diseases. End-of-life refers to the part of the disease trajectory where patients are likely to die within 12 months [14].

No single strategy has so far proven superior in optimising palliative care in general practice [15, 16]. A revised Danish guideline on palliative care in general practice was published in 2014 [17], but a guideline in itself does not change the clinical practice [18]. Continuing medical education (CME) meetings have shown to have a positive effect on changing the GPs’ attitudes concerning palliative care, but they seem to have little impact on the actual provision of care [15]. Electronic decision support (EDS) has improved guideline adherence in other areas (e.g. prescription of antibiotics) and changed clinical outcomes [18–23]. However, a Scottish study showed that GPs were reluctant to use EDS in end-of-life care as the term “palliative” was hard to apply to electronically identified patients because of the association with death [24]. Still, it is unknown whether a combination of CME and EDS could optimise end-of-life care provision among GPs.

The aim of this study was to develop and pilot-test an intervention consisting of a CME meeting and EDS to support the end-of-life care in general practice for patients with cancer or COPD.

## Methods

This study describes the development of a complex intervention in general practice based on the recommendations of the Medical Research Council (MRC). The MRC guidelines included both recommendations for conducting the study and for reporting the results [25, 26]. The MRC framework suggests four phases in the development of a complex intervention. This study focuses on the first two phases; phase 1 focus on development of the intervention and includes evidence-based identification of *barriers* to GP provision of end-of-life care and perceived *facilitators* to change the clinical practice and the modelling of the intervention. Phase 2 is pilot-testing of the intervention.

To reduce the complexity of the intervention the overall phases in the MRC guideline and how we planned the steps will be presented in the method section. The result section will specify the content and the results of the different steps.

### Phase 1: development of the intervention

First part of the development was to *identify* barriers to end-of-life care and facilitators to clinical change in general practice. This was done using three different strategies.

Two narrative literature searches were performed: one focused barriers to end-of-life care and another on facilitators. The following medical databases were searched: biblioteket.dk, SweMed, PubMed, Embase, Cochrane Library, Cinahl and PsyhINFO.

#### Search terms

1. General practice OR General practitioners OR family practice OR family doctor AND palliative care OR palliative medicine OR end-of-life care OR terminal care
2. General practice OR general practitioners OR family practice OR family doctor AND clinical practice OR change of clinical practice OR intervention study OR continuing medical education

Reference lists were subsequently scrutinised for additional studies, and relevant articles were selected after reading the abstracts.

Secondly, AKW performed unstructured individual interviews with three GPs with a special interest in end-of-life care. The aim of these interviews was not to achieve saturation of data but to test and culturally adapt the established knowledge on barriers and facilitators from the literature to a Danish clinical setting to help choosing the right focus.

Thirdly, the findings were discussed within the research group (constituted of the authors) drawing on own research and clinical experiences.

Second part of the development phase was the *modelling* of the intervention.

Based on the identified evidence base, the research group selected a number of barriers to address from a perspective of importance and barriers possible to address. Furthermore, facilitators to increase the effect of the intervention were selected. A multifaceted approach with a tailored intervention was chosen [27, 28] with two components, which complemented each other: a CME meeting and an EDS. The CME meeting was a one-time event allowing time to reflect and engage with colleagues, whereas the EDS continuously provided contextually relevant evidence-based information without interaction with peers.

Hence, two working groups, including stakeholders with CME experience in general practice, were appointed to assist in designing the components. The group developing the CME meeting comprised of seven participants: the research group (including an oncologist, two researchers with special interest in general practice and a palliative care specialist), one GP responsible for a regional CME, and two academic coordinators for CMEs targeting GPs in the region.

The EDS working group comprised of two GPs, the research group, and medical and technical staff from the Danish Quality Unit of General Practice (DAK-E). Two successive meetings were held during the development with participation from the GPs engaging in CME, administrative staff from all regions in Denmark, and a member of the research group (AKW). The technical development was carried out by DAK-E. The EDS was made using existing technology to ensure compatibility with all electronic patient record (EPR) systems in Danish general practices [3, 29].

### Phase 2: pilot-testing

In the pilot-testing, we adapted ideas and terms from the MRC guideline [26] and the process evaluation described by Grol et al. [18]. The purpose of process evaluation is to systematically assess the components in the intervention that could have an impact on the outcome of a pilot study. There is no standard process evaluation, but assessment is suggested to include: 1) the fidelity, 2) the quality and 3) the context of the intervention [18, 26]. We assessed the CME meeting and the EDS separately.

Degree of adherence to the blueprint and the reach of the intervention assessed the *fidelity*.

Adherence to the blueprint examines the extent to which the intervention components were delivered as intended, including whether development of the components succeeded and how well the components were implemented. To ensure adherence to blueprint in the CME meeting and delivery of similar content in each CME meeting, a test run was performed. All persons engaged in teaching at the CME meeting were present and received a copy of a detailed plan.

The implementation plan primarily included using existing newsletters which were sent to all GPs from two different senders. The Quality Unit for Cancer care in general practice in Central Region Denmark invited and reminded the GPs to participate in a CME meeting (free of charge) in their catchment area. The GPs were informed about the EDS through the regular DAK-E newsletter. Furthermore, the EDS was demonstrated at the CME meetings and briefly presented in a trade journal for Danish GPs [30].

The reach of the intervention was assessed by number of GPs attending CME meetings or signed up for the EDS compared to those who did either one or none of them. Background characteristics were retrieved for all GPs in the region to allow comparisons and to clarify if the intervention targeted specific subgroups of GPs i.e. younger GPs, urban GPs or female.

The *quality* of the CME meeting was investigated by using the attending GPs’ experiences of the meeting and the impact of the meeting on provision of end-of-life care. GPs’ experiences were assessed by using a mixed-methods approach carried out by an external evaluation unit in the Central Denmark Region [31]. The evaluation was done using questionnaires and interviews. After each meeting, a questionnaire was handed out to all participating GPs for themselves to fill in. The questionnaire consisted of seven items concerning benefits of attending the CME meeting, applied teaching methods, suggestions for improvements and if/how the CME meeting might affect their approach to end-of-life care in the future. Two of the items were answered on a 5-point Likert scale ranging from one to five and the remaining five questions were answered by free text comments (see Additional file 1 to see the questionnaire).

In addition to the questionnaire, three group interviews with fixed questions were conducted. Each interview was performed with a group of three GPs and carried out immediately after three of the six CME meetings (i.e. a total of nine interviewed GPs). The interviews focused on three topics: teaching methods, benefits from the CME meeting and possible improvements for future educational meetings. Each interview took approximately 15 min.

The short-term impact of the CME meeting on the GPs’ attitude was assessed by an email sent three months after the CME meeting to all participating GPs. They were asked: Have you changed anything in your approach to palliative care since the CME? (If yes: then what?; if no: then why not?).

To assess participants’ experience with the EDS, a postal questionnaire was planned to be send one year after the implementation to the GPs. The questionnaire contained items about relevance and functionality of the EDS. Furthermore, the specific function of the EDS that identified patients with potential palliative needs (more details are available in the results section), was to be adjusted retrospectively by using register-based data on deceased patients to possibly adjust the criterions for identification. These register-based data were also to be compared to how often the GPs ticked the pop-up window as irrelevant. Finally, as a proxy for usage of the EDS, data on how many times a pop-up was opened by the individual GP were to be retrieved.

The impact of the intervention on provision of end-of-life care was to be fully assessed after one year. The assessment would focus on patient-related outcomes on practice level, e.g. number of terminal declarations (a declaration releasing medical reimbursement for end-of-life care), frequency of prescription of anticipatory medication used in the terminal phase and number of home deaths. These data were to be retrieved from national registers using the unique identification number of every Danish citizens and their exact linkage to a general practice. The figures were to be compared before and after the intervention.

Finally, the *context* of the intervention was assessed by focusing on elements that could facilitate or hamper the effect of the intervention.

The overall context was the Danish health care system, which is tax financed and provides free access for all residents to health care services. More than 98% of the Danes are registered with a specific general practice, and the GPs are responsible for the health care provision to their listed patients [3]. If symptom relief or problem solving is too complex or not possible in primary care, the GPs can get advice from specialists or refer to specialist treatment [3]. The GPs are remunerated by Danish Regions according to a nationally negotiated scheme. Continuing medical education (CME) was not compulsory for Danish GPs until 2015, but they could receive remuneration for five days a year to cover education expenses and loss of earnings [32]. This study was performed in the spring 2014 in the Central Denmark Region with 843 GPs organized in 407 practices covering a population of approx. 1.3 million inhabitants.

### Data collection

Concerning the CME meeting, all participating GPs were registered (provider number, qualified GP/trainee/other, municipality, place of participation) upon arrival at the meeting for evaluation of the implementation of the CME meeting. GP characteristics (provider number, gender, number of GPs in different areas) were retrieved from the Central Denmark Region to compare participating and non-participating GPs. The external evaluator, who carried out the evaluation of the CME meeting, registered the answers on a Likert scale and collected the answers to the open questions in full wording for each topic and each meeting separately. The interviews were recorded and summarized in themes grouped according to the three overall topics (teaching methods, benefits from the CME meeting and possible improvements of the meeting) for each of the three interviews and the points were illustrated with typical statements from the interviewed GPs.

Data about EDS sign-up were retrieved from the DAK-E database. The patient-related data we had planned to use to fully assess the intervention was to be retrieved from national registers and DAK-E database.

### Analysis

Descriptive analysis regarding the attending GPs and their answers to the questionnaires were made. The Likert Scale answers were presented as percentages. The remaining five items with open answers were described qualitatively on the basis of trends and frequency of issues within each topic. Excel® was used for the descriptive statistics. As the analysis of the interview data were performed by an external evaluator no further information can be provided.

## Results

### Phase 1: the development phase

#### The barriers chosen to address by the research group based on the evidence base, unstructured interviews and experience were

Firstly, lack of identification of patients in end-of-life phase, especially for patients with non-malignant diseases [10]. Most clinicians tend to overestimate the remaining life span [33–35], which may compromise timely provision of end-of-life care. Different disease trajectories create different challenges for the GPs in the recognition of end-of-life issues. This could be one reason why patients with COPD tend to get less end-of-life care although their symptoms and prognosis are comparable to those of patients with lung cancer [11, 36, 37]. Secondly, variation in skills and knowledge among GPs concerning the provision of end-of-life care [38–40]. One issue is that some GPs tend to avoid confronting patients and relatives with end-of-life issues, whereas patients and relatives expect the GPs to take such initiatives, to be proactive, and assume the keyworker role in palliative care [40–42].

The facilitors chosen, by the research group, to be used in the intervention to emphasize the effect of the different components are listed in Table 1.

**Table 1:** Facilitators supporting the effect of a CME meeting

Case-based teaching [23]	
Guidance rather than orders [43]	
Educational meetings in small groups [13]	
Engaging with peers [13, 23, 43, 44]	
Active participation [13, 23, 43, 44]	
Sharing experiences with end–of-life care [13, 23, 43, 44]	
Involving opinion leaders [13]	
Encounters with specialist [13, 43]	

### The modelling

The framing of the CME meeting was based on adult learning theory with a problem-based approach to emphasise the relevance to clinical work [28]. The framing paid attention to the independent way in which most GPs work and their preferences for guidance [43, 44]. The content of the CME meeting was supported by research findings on GPs and end-of-life care, and an updated national guideline on palliative care for general practice published by the Danish College of General Practitioners (Table 2) [17].

**Table 2:** Programme and content of the CME meeting about palliative care

Time	Curriculum covered in each meeting
4.30–5.10 pm	What is palliative care? - Definition and changes in the understanding of palliative care. Focus on end- of-life care - Disease trajectories and the challenges in identifying when end-of-life care is needed - Discussion of patient case: (short film)
5.25–6.00 pm	What are the patients’ palliative needs? - Results from a Danish survey among palliative patients - Discussion of two patient cases (short films)
6.30–6.45 pm	Presentation of the local palliative team by the palliative physician
6.45–7.35 pm	Medical skills and practicalities - Prescription of just-in-case^a^ box, terminal declaration^b^, use of EDS, etc.
7.45–8.00 pm	Local support to patients and relatives - Which alternatives does the GP have? Who else can help and support?

^a^anticipatory medicine

^b^declaration releasing medical reimbursement for end-of-life care

The case-based teaching alternated between lectures and discussions. Three short films were produced for the meetings based on research findings to cover the topics in the meetings (Table 2). The films were used to facilitate the discussions between the GPs. Engaging with peers, participating actively and sharing own experiences with end-of-life care all aimed at increasing the effect of a CME meeting [18, 28, 44, 45]. GPs with special interest in end-of-life care were teaching together with a local palliative care specialist. Six identical CME meetings were held throughout the region based on the catchment area of the specialist palliative care teams.

**Fig. 1:**
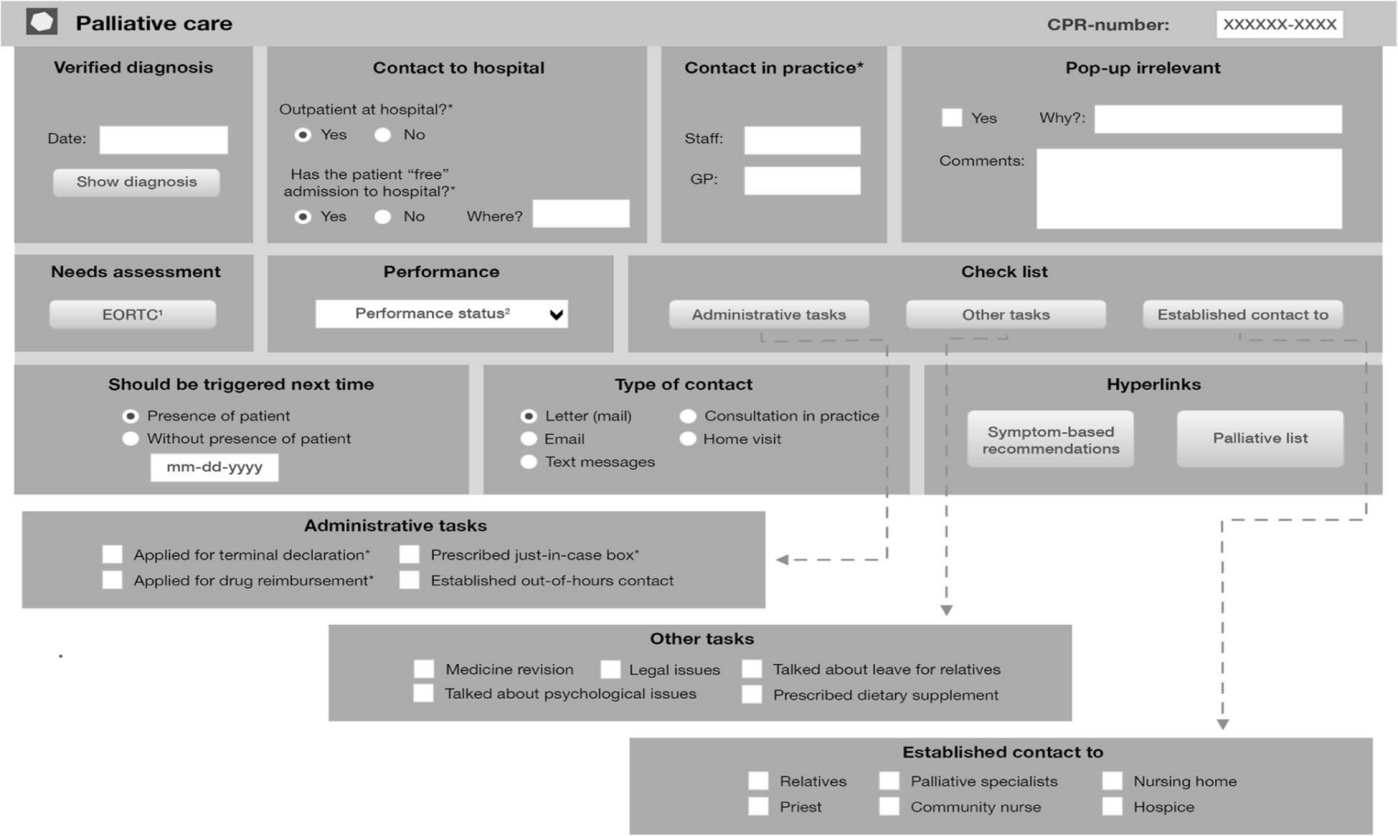
The EDS pop-up window generated in the medical records to be filled in by GP. 1: Directly linked to the EORTC QLQ-C15-PAL [55] in the palliative guideline [17]: ready to print and hand out to the patient. 2: ECOG Performance Status [56]. * The information is automatically transferred to the palliative list

**Fig. 2:**
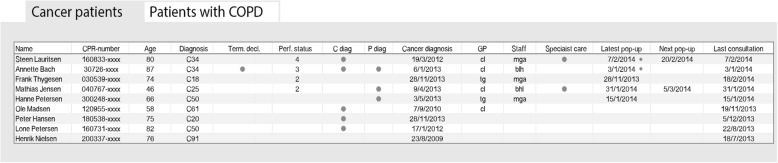
The list of all patients with palliative needs in the practice divided into patients with cancer and COPD, respectively. The tab for COPD contains additional information on smoking status, number of exacerbations within the last year and MRC breathlessness score. All information shown in the figure is made up for the figure and not based on real data. CPR number: Personal identification number allocated to every Danish citizen. Diagnosis: The cancer diagnosis (ICD 10). When the cursor marks the diagnosis, it is written in words. Term.decl: Terminal declaration. Data retrieved from the pop-up window. Perf. Status: ECOG performance status [56]. Data retrieved from the pop-up window. C and P diag: Comorbidities and psychiatric comorbidities; a dot means that the patient is registered with comorbidity (written in text when the cursor is dragged to the dot). Data retrieved automatically from the EPR. GP/staff: The patient’s contact GP/staff in the practice(s). Data retrieved from the pop-up window. Specialist care: The patient receives specialist palliative care. Data retrieved from the pop-up window. Latest pop-up window: A marker indicates that a note has been left by the GP/staff in the pop-up window (can be read when the cursor is dragged to the dot)

The final EDS consisted of two connected parts: i) a pop-up window in the patient’s medical record (Fig. 1: *The EDS pop-up window generated in the medical records to be filled in by GP*) and ii) a list of patients with end-of-life needs and key elements in their care (Fig. 2: *The list of all patients with palliative needs in the practice divided into patients with cancer and COPD, respectively*).

The pop-up window in the patient’s medical record had four functions: an identifier of the patient’s potential end-of-life needs, a reminder to the GP of the patients and actions to take, a provider of medical advice, and a checklist of palliative tasks to consider during the end-of-life trajectory (Fig. 1).

To serve as an identifier, the pop-up window was triggered on the first time the GP opened the patient’s medical record if at least one of the following codes were registered in the electronic patient record (EPR): diagnosis of malignancy, palliative diagnosis or COPD with either MRC dyspnoea scale = 5 [46], body mass index < 18 or forced expiratory volume in 1 s < 30 (see Additional file 2 for exact list of diagnosis). The trigger diagnoses were chosen from existing identification tools [47–49] and available patient information in the EPR. The aim was to identify patients with an estimated remaining life span of 12 months or less. The GP was asked to confirm if the patient was in the end-of-life phase. Additionally, the GP had to indicate the subsequent trigger: either next time the patient would be present in the GP practice or a specific date. This procedure was chosen so that the pop-up could work as a reminder, thereby making it easier for the GP to assume a proactive approach.

The pop-up window provided symptom-specific medical suggestions based on the recommendations from the national clinical guideline [17]. This function of the pop-up was integrated into the medicine module in the EPR to allow quick comparison between medical recommendations and prescribed medications. This easy access to medical advice was made to counterbalance any inadequate medical skills or doubts concerning end-of-life care among the GPs.

The checklist functionality in the pop-up window showed important issues to consider at some point of the palliative trajectory, e.g. making a terminal declaration, prescription of “in-case” box (anticipatory medicine), or registering performance status (Fig. 1). The checklist was linked directly to printable forms and assessment tools to minimize the time spent on administration and paper work. The checklists were also linked directly to the corresponding section in the online version of the national clinical guideline [17], where each issue was explained in detail.

The other part of the EDS was the list of the all patients identified by the GP as being in the end-of-life phase; this list was designed to help organise the care and promote a proactive approach. Two entries into the list were possible: either if the GP filled in anything in the pop-up (apart from ‘irrelevant’) or registered the patient as being in end of life by applying the International Classification of Primary Care diagnosis code “A99” in the EPR. The list showed key elements in end-of-life care (e.g. receiving specialised palliative care, palliative phase, date for next contact) for each patient listed (Fig. 2). If the GP was uncertain about a heading, the cursor could be dragged to the heading and an explanation would appear.

The information about key elements was automatically retrieved from data in the pop-up window and shown on the list using colour codes and simple explanations. The list was divided into two tabs to allow different information for different patient groups: one for cancer, one for COPD (Fig. 2). Another use of the list was to support the GPs in monitoring the clinical work. Additional suggestions as to how to use the data for this purpose were available for the GPs at the homepage of DAK-E [29].

### Phase 2: pilot-testing of intervention

#### The fidelity

The fidelity (adherence to blueprint and reach) was examined for the two components separately. All CME meetings were carried out as planned according to the schedule and the script. Hence, the adherence to the blueprint of the CME meeting was high. The EDS was developed as intended, and all the functionalities were integrated in the EPR. However, the development was delayed with regard to the implementation, as it was not ready for use at the time of the CME meetings, which may have decreased the possibility of synergistic effect. At the time of the intervention there was a discussion of legal issues concerning data collection from GPs in Denmark in general leading to a shutdown of most data collection from GPs in Denmark. Despite this was a national issue unrelated to the present project, we had to shut down the EDS untimely, as it was based on these data. Hence, the EDS was only running for a short time. The functionality showed high adherence to the blueprint but the implementation had low adherence.

The six CME meetings were attended by a total of 120 GPs, which is 14.2% of the 843 invited GPs. A relatively higher proportion of female GPs attended in comparison with the gender distribution among all GPs in the Central Denmark Region (Table 3).

**Table 3:** Characteristics of the CME-attending GPs and all GPs in the Central Denmark Region

	Participants^a^	GPs in the Central Denmark Region
GPs (n(%))	120 (100)	843 (100.0)
Age, (median iqr), years	54 (15)	54(14.4)
Gender, (n(%))		
Male	43 (35.8)	434 (51.5)
Female	77 (64.2)	409 (48.5)
Place of meeting, (n,(%))		
Viborg	8 (6.6)	84 (10.0)
Horsens	18 (15.0)	136 (16.1)
Silkeborg	15 (12.5)	67 (8.0)
Herning	25 (20.8)	185 (22.0)
Randers	25 (20.8)	144 (17.1)
Aarhus	29 (24.2)	223 (26.5)
Unknown	–	4 (0.5)

^a^ Additional 19 persons participated: 15 GP trainees, 3 nurses, or 2 other health care persons

The EDS reached fewer of the 843 invited GPs as only 50 GPs (5.9%) signed up. We could not retrieve information about the GPs who signed up for the EDS due to the above-mentioned untimely shutdown of the system. The overall reach of the intervention was low, which compromises the fidelity.

#### Quality of CME meeting

**Fig. 3:**
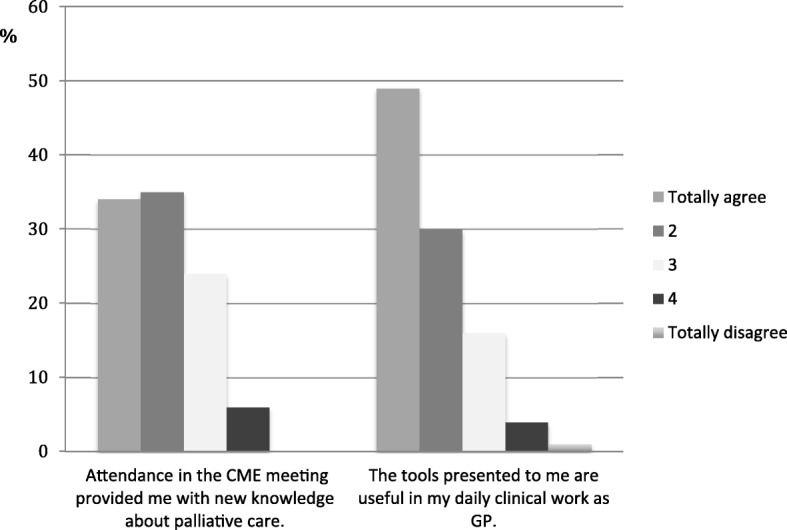
The distribution (% of responses (*n* = 115)) of GPs’ self-reported usefulness of attending the CME and the demonstrated tools. Made by the Committee for Quality Improvement and Continuing Medical Education in the Central Denmark Region [31] as a part of the evaluation of the CME sessions

In total, 115 (95%) GPs answered the questionnaire about the quality of the CME meeting. The CME meeting was well received by the attending GPs; overall they reported that they benefited from participating and gained new knowledge (Fig. 3: *The distribution (% of responses (n = 115)) of GPs’ self-reported usefulness of attending the CME and the demonstrated tools*). This was further explored in the interviews as the informants all stated to have benefited from the participation, independent of pre-existing familiarity with palliative care.

The presentation of palliative tools and the instructions on how to complete a request for drug reimbursement due to terminal illness was regarded as useful and appreciated (Fig. 3). One statement from the interviewed GPs illustrated this:

*“Then you have something to bring back to the practice and show to the others”.*

The teaching style with a mix of lectures and discussion in smaller groups worked well according to the GPs’ questionnaire responses.

The interviewed GPs rated the content of the meeting to be at a high level and found that the teachers were updated in the field of palliative medicine. They also emphasised that teaching by alternating persons and the mix of lectures and discussions with peers worked well. Illustrated by another statement from one of the interviewed GPs:

*“You sit and start thinking, and then it is nice to have the opportunity to share the thoughts with people around you”.*

Potential improvements suggested both by the interviewed GPs and at the open questions in the questionnaire was: to give higher priority to demonstration of the practical skills, extend the duration of the meeting to allow more peer discussions, more case-based work, and ensure more time for interaction between the palliative specialist and the GPs.

Three areas relating to end-of-life care also emerged as new to the GPs from the open questions in the questionnaire: they obtained a broader understanding of end-of-life care and realized that it also embraces other patient groups than cancer patients, increased their awareness of their proactive role, and directed more attention towards patients with potential palliative needs. Furthermore, the importance of using a more systematic approach and organising end-of-life care at practice level was highlighted.

The same points were made by the interviewed illustrated by this statement:

*“We might need to enter the playing field and not wait for the patients to come to us, right?”*

The CME meeting succeeded in addressing the main barriers. Yet, several issues that were raised at the CME meetings (e.g. awareness of the relatives’ needs and the importance of symptom screening) were not brought up as new insight in either the questionnaire of the interviews as new areas of awareness.

#### Impact of CME meeting

In total, 29 (25%) GPs participated in the three-month questionnaire evaluation. A fourth of the GPs stated specifically that they had adapted a more proactive approach to end-of-life care. Furthermore, they reported to have obtained increased awareness about palliative needs among patients with non-malignant diseases. Three of the 29 GPs (10%) stated that they had no patients with palliative needs after the CME meeting.

#### Impact of EDS

Due to the early shutdown of the EDS, we could not evaluate its impact. As the EDS was demonstrated on the CME meeting, many GPs made unprompted positive comments about the EDS in both the questionnaire and the interview. The GPs stated that they looked forward to using the EDS, and their immediate impression was that it would be a helpful tool, illustrated by this interviewed GP:

*“I will look forward to using the EDS which is on its way – it seems very applicable”.*

#### The context of the intervention

One specific event could in the context have hampered the effect. At the time of the implementation of the intervention, there was a nationwide disagreement between the GPs and Danish Regions. This might have made some GPs reluctant to participate as the regional administration, i.e. the Central Denmark Region, was involved in establishing the CME meeting. This connection was unintended as the staffs from the Central Denmark Region were involved exclusively to ensure high quality of the CME. The CME meeting was approved by the Public Health and Quality Improvement in the Central Denmark Region to allow remuneration of the attending GPs.

## Discussion

### Main findings

It was possible to model the intervention to address identified barriers to end-of-life care in general practice and integrated facilitators to enhance the effect of the intervention. Although the participation rate was only 15%, the pilot-testing showed that the CME meeting was well received among the attending GPs. The meeting had an immediate impact on the GPs and addressed the identified barriers, which suggests high quality. Evaluation of the EDS could not be performed due to early shutdown. The process evaluation of the pilot-test revealed a need to look further into how the intervention in full scale could be designed to reach more GPs.

### Comparison with other studies

One barrier we intended to address in both the CME meeting and the EDS was identification of patients with palliative needs [33, 50, 51]. A Dutch intervention by Thoosen et al. found that patients with palliative needs identified by their GP had more contact with their GP, less hospital admissions, and were more likely to die at home than not identified patients [52]. These findings underline the importance of the GP’s awareness and identification of patients with palliative needs. In the study by Thoosen et al., the GPs had to apply an identification tool (RADPAC) by going through their patients manually. Hence, the identification was still dependent on the GP’s awareness of palliative needs. Mason et al. made a computerised tool to identify patients with deteriorating health due to advanced conditions [24]. They found that some GPs were reluctant to register the computer-identified patients as “palliative” due to associations to death. The resistance against using the term “palliative care” earlier in the disease trajectory compromised the effect of the tool. This underlines the need for a change in attitude alongside the implementation of an EDS.

The attendance rate in the CME meeting was low in our study compared to other Danish studies. A disease management programme in 2010 in the same region had an attendance rate of 69% [53]. However, these GPs were remunerated for participating, and the programme formed part of a regional initiative aiming to prioritise and optimise chronic care management. Another Danish study with 1-h CME meetings in 2012 focusing on lung cancer diagnosis had an attendance rate of 49% [54]. The low attendance rate in this study could have several explanations: lack of interest, no need for education in end-of-life care, bad timing of the intervention, or poor implementation of the CME meeting.

The low attendance rate in our study is unlikely to reflect a lack of need for education in end-of-life care. A prior Danish study identified a need of improvement of palliative care skills and a lack of confidence in providing end-of-life care among GPs [10, 38]. Furthermore, a British study reported that most GPs wanted training focusing on different care issues when asked about their educational preferences in palliative care [39]. The timing of the intervention may have adversely affected the attendance rate due to the disagreement between Danish Regions and the GPs. Finally, despite that the newsletter is the common way to invite GPs to participate in continuing medical education, we do not know if they actually read the invitation. Hence, the effect of different methods of inviting the GPs should be investigated in future studies.

### Clinical implications and future perspectives

The increased longevity in the population may result in rising incidence of cancer and more terminally ill patients with non-malignant disease. Most of these patients wish to be cared for at home. Hence, there is a growing need for GPs who are skilled and confident in providing palliative care.

The evaluation of the intervention revealed increased awareness among GPs of potential palliative needs in other patient groups than cancer patients. The findings also suggest that the GPs should take a more proactive role and organise the care. Although the participating GPs suggested higher prioritisation of demonstration of practical skills in general in the CME, we recommend maintaining the current balance between practice and theory. The reason for this is increased awareness and broadening of understanding of end-of-life care together with improved skills are prerequisites for optimising care, which would benefit all groups of patients.

As the CME meeting was well received by the GPs and addressed the main barriers to end-of-life care, it is ready to be used in a full-scale study assessing the effect of the intervention on patient-related outcomes. However, the EDS needs to be evaluated before used in full-scale study. Furthermore, it could be beneficial to assess the implementation itself, as the participation rate was low in the set-up tested in this study.

### Strengths within the study

One of the strengths of the study was the systematic development of the complex intervention in accordance with the MRC guideline [46], which facilitated integration of evaluation in the design. This allowed analyses of the different steps and elements of the complex intervention and enabled us to investigate if the different components in the intervention worked as intended. This approach generally improves the applicability of tailored interventions to other settings as it makes it easier to adapt relevant components.

Another strength of the study was the inclusion of stakeholders from an early stage of the modelling of the intervention; this increased the applicability and facilitated the implementation. The reach of the intervention was evaluated using register data, which allowed comparisons between attending GPs and all GPs in the region. The external evaluation carried out in the CME meeting reduced the risk of bias, especially with regard to the interviews after the meetings. On the other hand important information might be lost from the interviews as the data was interpreted by an external evaluator. However, the external evaluator had participated in a meeting prior to the evaluation to ensure appropriate focus. The use of questionnaires for evaluation of the CME allowed us to assess both the immediate and the three-month self-perceived effect of the CME meeting on the GPs. However, the low participation in the follow-up compromised the generalisability of the three-month effect.

### Limitations within the study

The use of narrative literature search to identify the barriers to end-of-life care introduced a risk of missing information, as the search was not exhaustive However, as the aim with the literature search only was to investigate barriers this was not a methodological concern in this study.

A limitation of using the guideline was the lack of standard process evaluation [18].

For the impact of the intervention the early shutdown of the EDS was a major limitation. This prevented evaluation of the reach, the participants’ experience, and the quality and of the EDS. Another limitation of the study was the low attendance rate at the CME meetings and the lack of possibility to examine attendance further in the current design.

## Conclusion

A complex intervention consisting of CME meetings and EDS to aid GPs provide better end-of-life care was developed using MRC guidelines and current evidence. The evaluation of the pilot-test showed overall appreciation of the CME meetings, which addressed identified barriers to providing care. The EDS was shut down early and needs further evaluation before examining the whole intervention in a larger study, where evaluation could be based on patient-related outcomes and the impact on end-of-life care.

## Additional files

Additional file 1: Questionnaire used to evaluate the continuing medical education meeting. (DOCX 19 kb)

Additional file 2: List of ICPC and ICD-10 codes that prompted the pop-up window in the patient’s medical record. (DOCX 47 kb)

## Abbreviations

AKW: Anna Kirstine Winthereik (part of research group)
CME: Continuing Medical Education
COPD: Chronic Obstructive Pulmonary Disease
EDS: Electronic decision support
EPR: Electronic patient records
GP(s): General practitioner(s)
ICD-10: International Classification of Diseases, version 10
MRC: Medical Research Council
