# Supplementary material for: Development, modelling, and pilot testing of a complex intervention to support end-of-life care provided by Danish general practitioners
Source: BMC Fam Pract. 2018 Jun 20;19:91. doi: 10.1186/s12875-018-0774-x (PMC6011239; doi:10.1186/s12875-018-0774-x)
Supplement: Supplementary file 1 — Questionnaire used to evaluate the continuing medical education meeting. (DOCX 19 kb) [file 12875_2018_774_MOESM1_ESM.docx]

Questionnaire to GPs after their participation in the CME meeting

1: Attendance in the CME meeting provided me with new knowledge about palliative care (please mark the most appropriate response):

Totally agree 1 2 3 4 5 Totally disagree

2: The tools presented to me are useful in my daily clinical work as GP (please mark the most appropriate response):

Totally agree 1 2 3 4 5 Totally disagree

3: What did you get out of participating in the CME meeting? (please state which)

____________________________________________________________________________

____________________________________________________________________________

4: How did you benefit from participating in the CME meeting? (please state how)

____________________________________________________________________________

____________________________________________________________________________

5: What do you think about the applied teaching methods?

____________________________________________________________________________

____________________________________________________________________________

6: What could have improved your benefit from participating? (please state what)

____________________________________________________________________________

____________________________________________________________________________

7: On the basis of this meeting I intend to change the following in my approach to palliative care (please state what) ____________________________________________________________________________

____________________________________________________________________________

Made by the Committee for Quality Improvement and Continuing Medical Education in the Central Denmark Region [31] as a part of the evaluation of the CME sessions. Translated by Anna K. Winthereik
