# Supplementary material for: Development, modelling, and pilot testing of a complex intervention to support end-of-life care provided by Danish general practitioners
Source: BMC Fam Pract. 2018 Jun 20;19:91. doi: 10.1186/s12875-018-0774-x (PMC6011239; doi:10.1186/s12875-018-0774-x)
Supplement: Supplementary file 2 — List of ICPC and ICD-10 codes that prompted the pop-up window in the patient’s medical record. (DOCX 47 kb) [file 12875_2018_774_MOESM2_ESM.docx]

**Additional file 2**

ICPC [58, 59] and ICD-10 [60] codes that prompted the pop-up window in the patient’s medical record were retrieved from discharge summaries (ICD-10) or the GP’s medical record (ICPC):

ICPC codes:

Malignancies:

A79, B72, B73, B74, D74, D75, D76, D77, L71, N74, R84, R85, S77, T71, U75, U76, U77, W72, X75, X76, X77, Y77, Y78

Chronic obstructive pulmonary disease:

R95: MRC5, BMI<18 or FEV1<30 is registered in the patient’s medical record.

A99: One of the above ICPC codes is listed.

ICD-10 codes:

DZ515, DC76, DC77, DC78, DC79, DC80 (all sub-codes are included)
